# Accuracy of magnetic resonance imaging in defining dentate line in anal fistula

**DOI:** 10.1186/s12880-022-00927-x

**Published:** 2022-11-18

**Authors:** Xiuxiang Liu, Zhenchang Wang, Hua Ren, Zheng Wang, Jing Li

**Affiliations:** 1 Department of Radiology, Xiyuan Hospital, China Academy of Chinese Medical Sciences, No. 1, Road. Xiyuancaochang, District Haidian, Beijing, 100091 China; 2 Department of Radiology, Beijing Friendship Hospital, Capital Medical University, Beijing, China

**Keywords:** Anal canal, Dentate line, Magnetic resonance imaging, Anal fistula

## Abstract

**Purpose:**

To retrospectively assess the accuracy of magnetic resonance imaging (MRI) in defining dentate line in anal fistula.

**Materials and methods:**

Seventy patients with anal fistulas were assessed by dynamic contrast-enhanced MRI. The distance from the dentate line to the anal verge for all patients was measured by MRI. To mitigate interference, 35 patients with anal fistulas whose internal openings were located on the dentate line were excluded from this study. Two observers independently judged the positional relationship between the internal opening and the MRI-defined dentate line, and compared with the results observed by surgeon to assess the accuracy.

**Results:**

The distance between the MRI-defined dentate line and the anal verge depended on the location of the internal opening and the morphology of the anal canal mucosa. The distance based on the location the internal opening and the morphology of the anal canal mucosa was 18.2 ± 8.1 mm and 20.0 ± 5.3 mm on oblique coronal T2WI, respectively. Compared with the results observed by the surgeon, the accuracy of evaluating the positional relationship between the internal opening and the dentate line from the morphology of the anal canal mucosa on MRI exceeded 89.9%. Taking 18.2–20.0 mm as the distance between the dentate line and the anal verge on the MRI image, the accuracy of evaluating the relationship between the position of the internal opening and the dentate line was over 85.7%. Considering both the dentate line and the anal canal mucosa, the accuracy of evaluating the relationship between the internal opening and the dentate line was over 91.5%. The results of MRI-defined dentate line were in good agreement with the results of intraoperative surgeon evaluation, and the κ values were 0.70, 0.63, and 0.78, respectively.

**Conclusion:**

MRI has high accuracy in defining the dentate line in anal fistulas.

## Introduction

MR images cannot show the dentate line of the anal canal, but in clinical work, it is the most significant anatomical landmark of the anal canal. For example, for low rectal cancer, the best anatomical landmark is the dentate line in the formulation of sphincter sparing resection [1]. The classification and removal of hemorrhoids are usually determined by the dentate line [2]. Surgeons are also particularly concerned about the positional relationship between the internal opening and the dentate line when performing anal fistula surgery [3]. In the current clinical work, the MRI report of the radiology department usually lacks a description of the positional relationship between the internal opening and the dentate line, which limits the preparation of the surgeon's preoperative diagnosis and treatment plan.

The anal canal (also known as surgical anal canal) extends from the anorectal junction to the anal verge, usually 4–5 cm in length for the adult [4]. The length of anatomical anal canal from the dentate line to the anal verge is 2–3 cm [5]. Some researchers believe that if the anal canal was divided into three equal parts, the dentate line would be located at the junction of the upper third and the lower two thirds [6]. However, other researchers argue that the dentate line lies on the plane between the deep and superficial parts of external anal sphincter [7]. Thus, more objective methods of locating the position of the dentate line on MRI are still needed. Usually, the distribution of epithelium above and below the dentate line are different. On the proctoscopy, the mucosa membrane of the anal canal dentate line shows a circumferential wavy mucosal fold. The anal epithelium above the dentate line is like the glandular epithelium of the rectal mucosa. It is composed of columnar cells, crypts and goblet cells and contains 8–10 anal columns. The inner surface of the anal canal under the dentate line is distributed with squamous epithelium without a keratinized layer, which has a smooth appearance under a rectal microscope [4]. According to the different characteristics of the dentate line and the mucosal tissues of the inferior anal canal, as well as the position of the internal opening in patients with anal fistula, the distance from the dentate line to the anal verge can be indirectly calculated. Along this line of thinking, it is possible to speculate and measure the distance between the dentate line and the anal verge on MRI. As far as we know, there are few studies using MRI to measure the dentate lines. The aim of this study was to assess the accuracy and feasibility of preoperative MRI in predicting the distance between the dentate line and the anal verge.

## Materials and methods

### Patients

102 consecutive patients with anal fistula were identified from our PACS database. These patients were admitted from January 2019 to March 2021 and all underwent surgical treatment in the anorectal surgery department of Xiyuan Hospital China Academy of Chinese Medical Sciences. To select them, three criteria need to be met. First, it needs to perform a dynamic contrast-enhanced MRI before the operation. Second, operation must be completed within one week after MRI. Third, operation must be performed by the same group of surgeons. 32 postoperative patients who did not have surgery, or had a history of rectal cancer or anal cancer, or had motion artifacts in MRI images were excluded from this study. In the end, 70 patients with anal fistula were included in this study, including 61 males and 9 females, with an average age of 42.67 ± 12.98 years and a range of 24–73 years. Seventy patients had 77 internal openings (7 patients had two internal openings, and the rest had only one internal opening). The clinical data characteristics of patients are shown in Table 1.

**Table 1:** Observed data in patients with anal fistula (N = 70)

Characteristic	Observed date
Age (y) (mean ± SD)	
Male	43.3 ± 12.9
Female	38.6 ± 13.3
Sex (%)	
Male	61/70 (87.1)
Female	9/70 (12.9)
Internal opening (number) (%)	
≤ 1	63/70 (90)
≥ 2	7/70 (10)
The position of internal opening (%)*	
Above the dentate line	35/70 (50)
In the dentate line	42/70 (60)
Below the dentate line	0
External opening (%)	
≤ 1	66/70 (94.3)
≥ 2	8/70 (11.4)
Number of fistulous tracks (%)	
Single	66/70 (94.3)
Multiple	4/70 (5.7)

The number in brackets indicates percentage

*Intraoperative finding (considered as the gold standard)

### Methods

#### Magnetic resonance imaging

The MRI was performed using a 3T MRI scanner (GE Medical Systems, Discovery-MR750) with an 8-channel abdominal coil. We did not prepare bowel or intubation for the anal canal or fistula. During the MR examination, the patient was in a supine position, and the positioning line was aligned with the upper edge of the pubic symphysis. All patients underwent routine pelvic MRI (anal canal) and dynamic contrast-enhanced MRI (DCE-MRI). On pelvic MRI (anal canal) scan, the sagittal plane is acquired first, then oblique axial scanning is performed perpendicular to the long axis of the anal canal, and finally oblique coronal scanning is performed parallel to the long axis of the anal canal. MRI scan sequence including oblique transverse T1WI and T2WI, oblique coronal T2WI, sagittal T1WI and DCE-MRI (Table 2). Dynamic contrast-enhanced MRI imaging was performed for a total of 7 dynamics (1 prior and 6 after contrast injection) with a temporal resolution of 31 s. Gadoteridol was administered intravenously at a dose of 0.1 mmol/kg (0.2 ml/kg) of body weight through an 18-gauge intravenous catheter with an automated injection pump (Optistar Elite, Mallinckrodt, Hazelwood, Mo). Injection of contrast medium was followed by a bolus of 15 ml saline solution at 2 ml/s. Oblique coronal T2WI was obtained with the following parameters: repetition time (4070 ms), echo time (114.8 ms), flip angle (90°), bandwidth (Hz/pixel 183.3), slice thickness/gap (4.0/0.4 mm), number of slices (20), acquisition matrix (320 × 288), field of view (250 × 250 mm) and acquisition time (1:58 min:sec). Dynamic contrast-enhanced MRI was obtained with the following parameters: sequence type (T1-LAVA), acquisition plane (SPAIR axial), repetition time (4.5 ms), echo time (2.1 ms), flip angle (90°), bandwidth (Hz/pixel 142.9), slice thickness/gap (4.0/2.0 mm), number of slices (616), acquisition matrix (256 × 224), field of view (260 × 234 mm) and acquisition time (3:30 min:sec).

**Table 2:** MRI acquisition sequence and parameters

Type	T1WI	T2WI	T2WI	T2WI	DCE-MRI
	FSE	FSE	FSE	CUBE	T1-LAVA
Acquisition plane	Oblique transverse	Oblique transverse	Oblique coronal	Sagittal	FS + oblique transverse
Repetition time (ms)	785	5395	4070	2500	4.5
Echo time (ms)	8.4	139	114.8	133.6	2.1
Flip angle (°)	90	90	90	90	90
Bandwidth (Hz/pixel)	141.7	141.7	183.3	162.5	142.9
Echo train length	4	24	24	100	-
Slice thickness/gap (mm)	3/0.4	3/0.4	4/0.4	2/1	4.0/2.0
Number of slices	25	25	20	220	616
Acquisition matrix	288 × 256	228 × 256	320 × 288	320 × 256	256 × 224
Field of view (mm)	200 × 200	200 × 200	250 × 250	256 × 230	260 × 234
Acquisition time (min:sec)	2:25	2:27	1:58	4:41	3:30

### Image interpretation: evaluation of the MRI-defined dentate line

#### The method of locating the internal opening on MRI

**Fig. 1:**
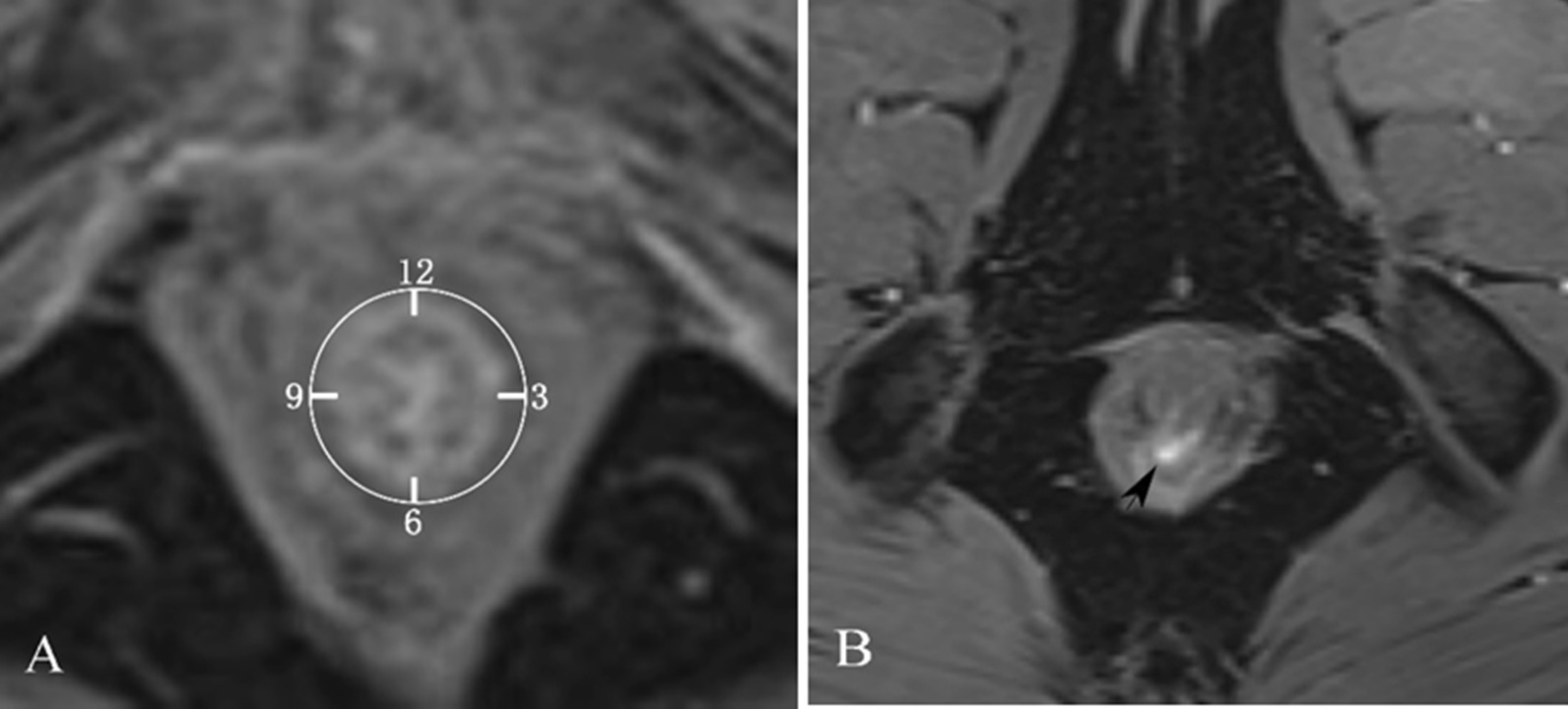
Schematic diagram of clock positioning method for anal fistula. **A** The front center is 12 points, the rear center is 6 points, the left center is 3 points, and the right center is 9 points. **B** The internal opening of anal fistula is at 6 o’clock (shown by the black arrow)

The location and number of anal fistula internal openings were determined on the images of the early arterial phase (46.5–77.5 s) of DCE in the oblique transverse section. The location of the internal opening is based on the clock positioning method of lithotomy position, the location and number of internal opening located by MRI were compared with those determined by intraoperative surgeons (Fig. 1). If there’s a discrepancy, the physicians were required to review the MRI images again, communicate and negotiate with the surgeons, find the reasons for the inconsistency, and finally unified the result.

#### Location of anal verge on MRI

**Fig. 2:**
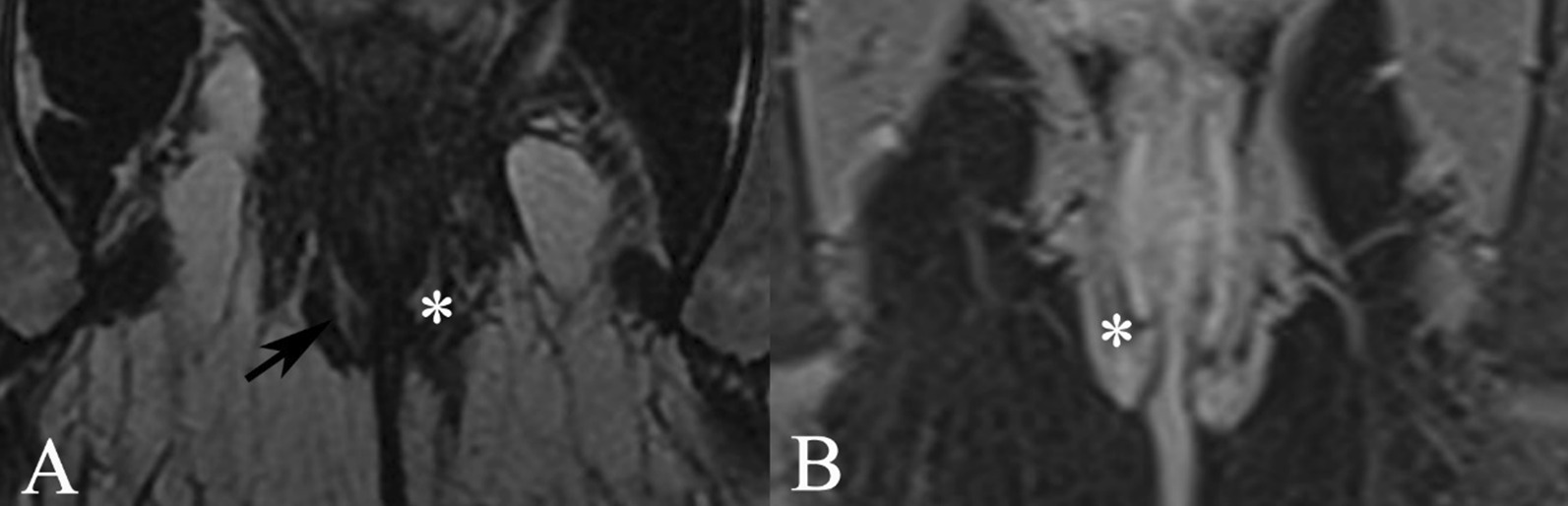
The location of the anal verge on oblique coronal T2WI. **A** oblique coronal FSE T2WI, compared with the signal of surrounding muscles, subcutaneous part of external anal sphincter is isointense (shown by black arrows); **B** oblique coronal enhanced T1WI, subcutaneous part of external anal sphincter is significantly enhanced uniformly, intersphincteric groove shown by asterisk

Referring to the study of Han et al. [8], the anal verge refers to the subcutaneous part of external anal sphincter. In this study, we defined the position of the lowest point of subcutaneous part of external anal sphincter on the oblique coronal T2WI as the anal verge. On oblique coronal T2WI, the subcutaneous part of external anal sphincter showed isointensity, and it was significantly enhanced uniformly after enhancement (Fig. 2).

### Distance measurement from dentate line to anal verge

#### Based on the location of the internal opening of the anal fistula

**Fig. 3:**
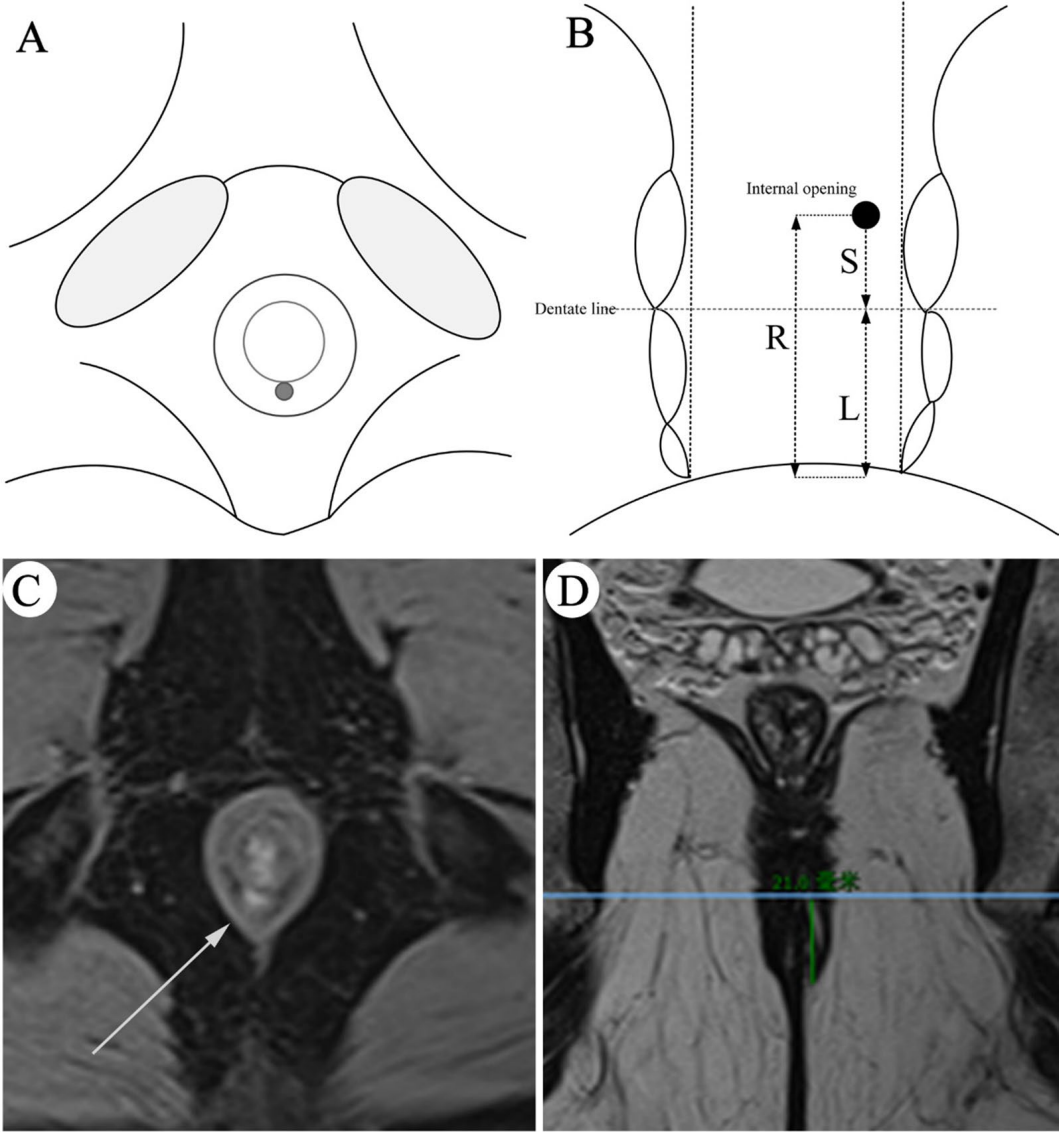
Anal fistula MRI-defined dentate line measurement. **A**, **B** are schematic diagrams of the determining dentate line on MRI, respectively. **A** The position of the internal opening of the cross section (arrow). B is a schematic diagram of a coronal image. Where S is the distance between the internal opening measured by the surgeon and the dentate line; R is the linear distance between the internal opening measured by the diagnostician and the anal verge (the underside of the external sphincter skin); and L is the distance between the dentate line and the anal verge (L = R–S). **C**, **D** are corresponding measurement methods on MRI images. **C** The dynamic contrast-enhanced fat-saturated T1-weighted MR image and **D** is coronary T2WI image, respectively

Physicians evaluated the position and number of internal opening by independent blind method on the early arterial phase of dynamic contrast-enhanced (46.5–77.5 s). Using MPR technology, this study first determined the position of the internal opening on the oblique coronal T2WI image, and then measured the linear distance (R) between the internal opening and the lowest point of subcutaneous part of external anal sphincter (anal verge) on the oblique coronal T2WI image, the surgeon estimated the distance between the internal opening and the dentate line by visual inspection (S) (Fig. 3). Finally, the distance (L) between the MRI-dentate line and the anal verge can be calculated by L = R–S.

#### Based on the morphology of the anal canal mucosa

**Fig. 4:**
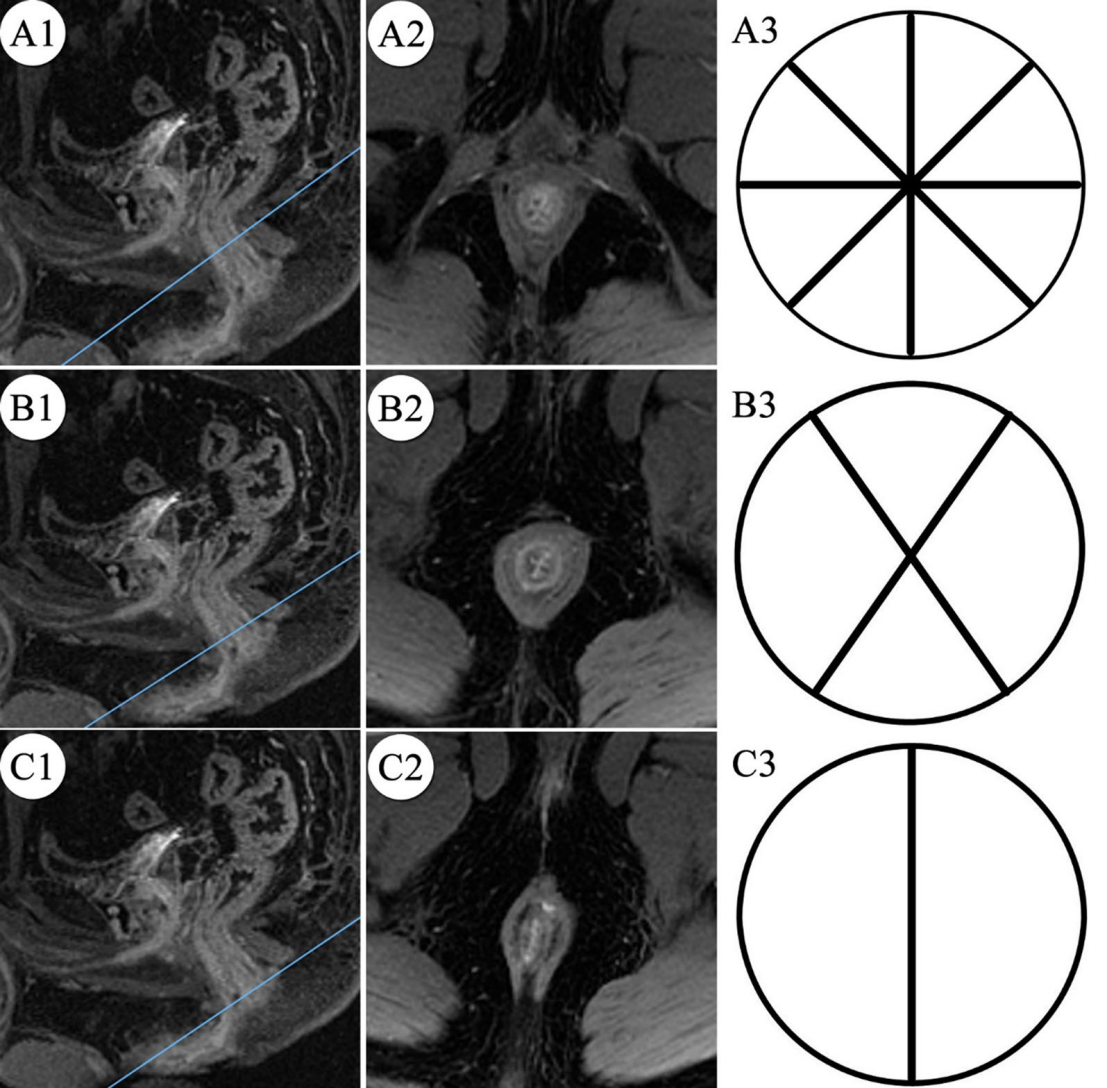
In the early arterial phase of dynamic contrast-enhanced fat-saturated T1-weighted MR images of the anal canal in 39-year-old male. **A**–**C** Axial images at the corresponding levels marked on the sagittal contrast-enhanced images and the morphology of mucosa was difference in anal canal according to location. **B** Represents the position of the dentate line and the morphology of the mucosa at the dentate line on MRI. A3, B3 and C3 are the corresponding schematic diagram of the anal mucosa, respectively. The black line represents anal canal mucosa

In the early arterial phase of dynamic contrast-enhanced MRI (46.5–77.5 s), the mucosa in the anal lumen varied in shape due to different positions. The upper anal mucosa was dense and columnar, the middle anal mucosa was irregular, sparse and flat, and the lower anal canal showed thick linear high signal. On the cross-sectional dynamic contrast-enhanced MRI, the dentate line coved the irregular, sparse and flat mucosa, and the images show “X” or “Y” (Fig. 4).

We used irregular flat and sparse mucosa in the anal canal as the sign of the dentate line on MRI, and excluded patients with anal fistula whose internal opening was at the level of the dentate line (affecting the interpretation of the dentate line on MRI). There were 35 cases of anal fistula patients whose internal opening was above the dentate line, including 29 males and 6 females, with an average age of 45.06 ± 12.24 years and a range of 25–68 years. Two physicians with 3 and 11 years of experience judged the position of the dentate line on MRI and measured the distance from the dentate line to the anal verge on MRI (also anatomical anal canal). The morphology of the anal canal mucosa and the position of the internal opening was determined on the oblique axis dynamic contrast-enhanced T1WI image of the early arterial phase, and the distance from the dentate line to the anal verge was measured on the T2WI oblique coronal position. The anal verge was the lowest point of the subcutaneous part of external anal sphincter. The evaluation results were compared with the intraoperative detection results of the surgeons. According to the mucosa judgment method and the internal opening judgment method, the anal fistula with the same evaluation results by two physicians were compared with the detection results of the intraoperative surgeons again.

#### Surgery

**Fig. 5:**
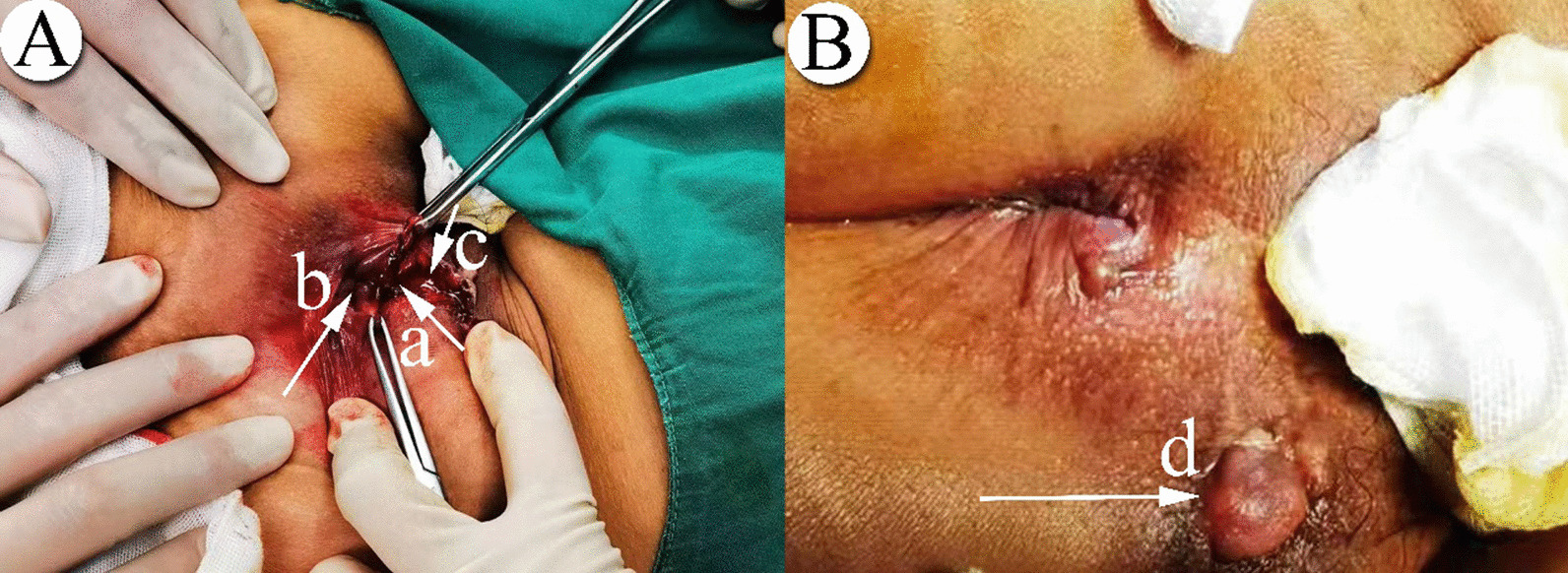
Images of a 34-year-old woman with anal fistula on the macroscopic picture. **A** Internal opening; **B** dentate line; **C** fistula; **D** external opening

All patients were operated by the same group (6 people) of anorectal surgeons with 8 years or more clinical experience in our hospital, and all operations were performed within one week after the completion of the MRI examination. The distance between anal verge and the dentate line was assessed macroscopically, and the distance was recorded by surgeon. Surgical evaluation was performed when the patient is in the lithotomy position or lateral decubitus, and received local anesthesia (perianal subcutaneous abscess or low intersphincteric abscess), sacral epidural or spinal anesthesia (high anal fistula, complex anal fistula, ischiorectal fossa abscess). During the operation, the surgeon recorded the position relationship between the internal opening and the dentate line, and visually measured the distance (S) between the internal opening and the dentate line, that is, the internal opening (a), dentate line (b), fistula (c), and external opening (d), respectively (Fig. 5). Fistula incision, fistula resection, and seton drainage are common surgical methods in our institution.

### Statistical analysis

According to the position of the internal opening and the morphology of the inner membrane of the anal canal, two physicians measured the distance between the dentate line and the anal verge (anatomical anal canal) using the mean ± standard deviation. The distance from the dentate line to the anal verge in men and women was measured by using mean ± standard. The positional relationship between the internal opening and the dentate line was determined by surgeon during the operation that used the intraoperative surgeon's evaluation of the positional relationship between the internal opening and the dentate line as the gold standard. Based on the mucosa judgment method and the internal opening judgment method, 2 physicians measured the distance from the dentate line to the anal verge, using 2 independent samples *t*-test and 2 independent samples Mann–Whitney U test, and we compared the consistency between groups by the Kappa test and Intraclass correlation coefficient. Two physicians judged the positional relationship between the internal opening and the dentate line, and calculated the sensitivity, specificity, accuracy, positive predictive value (PPV) and negative predictive value (NPV), and the consistency between the two physicians was measured by the κ value.

## Results

### The distance from the dentate line to the anal verge

On the oblique coronal T2WI, the distance from the dentate line to the anal verge according to the internal opening method measured by physician 1 was 18.2 ± 7.2 mm, which was 18.3 ± 7.4 mm in men and 17.1 ± 4.4 mm in women, respectively. The distance from the dentate line to the anal verge measured by physician 2 was 18.5 ± 8.2 mm, of which 18.1 ± 8.1 mm for men and 20.5 ± 9.7 mm for women, respectively (Table 3). The measurement results of physician 1 and physician 2 were averaged, and the final distance from the dentate line to the anal verge was 18.2 ± 8.1 mm, which was 18.3 ± 8.4 mm for men and 17.1 ± 6.1 mm for women, respectively.

**Table 3:** Distance from the dentate line to the anal verge in males and females according to the internal opening judgment method (mm)

	Male	Female	Mean ± SD
Physician 1	18.3 ± 7.4	17.1 ± 4.4	18.2 ± 7.2
Physician 2	18.1 ± 8.1	20.5 ± 9.7	18.5 ± 8.2

On oblique coronal T2WI, the distance from the dentate line to the anal verge based on the mucosa judgment method by physician 1 was 20.0 ± 4.3 mm, which was 20.0 ± 4.4 mm for male and 19.9 ± 4.1 mm for female, respectively. The distance from the dentate line to the anal verge measured by physician 2 was 20.0 ± 6.5 mm, of which 18.9 ± 6.0 mm for male and 25.2 ± 6.7 mm for female, respectively (Table 4). The average distance from the dentate line to the anal verge measured by the two physicians was 20.0 ± 5.3 mm, with 19.9 ± 6.0 mm for male and 20.0 ± 5.2 mm for female, respectively.

**Table 4:** Distance from the dentate line to the anal verge in males and females according to the mucosa judgment method (mm)

	Male	Female	Mean ± SD
Physician 1	20.0 ± 4.4	19.9 ± 4.1	20.0 ± 4.3
Physician 2	18.1 ± 8.1	25.2 ± 6.7	20.0 ± 6.5

### Comparison of the distance from the dentate line to the anal verge

According to the internal opening judgment method, the distances from the dentate line to the anal verge measured by physician 1 and 2 were 18.2 ± 7.2 mm and 18.5 ± 8.5 mm, respectively. There was no significant difference in the measurement results between the two physicians (*P* = 0.057). Good consistency between the two physicians was observed from this study (ICC = 0.55, *P* = 0.001). Based on the mucosal judgment method, the distances from the dentate line to the anal verge measured by physician 1 and 2 were 20.0 ± 4.3 mm and 20.0 ± 6.5 mm, respectively. There was also no significant difference in the results of the two physicians, and its consistency was good (ICC = 0.32, *P* = 0.03) (Table 5).

**Table 5:** The distance from the dentate line to the anal verge (mm)

	Physician 1	Physician 2	Mean ± SD	T value	*P*	ICC	*P*
Mucosal method (N = 35)	20.0 ± 4.3	20.0 ± 6.5	20.0 ± 5.2	0.244	0.144	0.32	0.03
Internal opening method (N = 77)	18.2 ± 7.2	18.5 ± 8.5	18.2 ± 8.1	0.071	0.057	0.55	0.001

Furthermore, according to the mucosal judgment method, there were 29 male patients with anal fistula and 6 female patients with anal fistula. The distance from the dentate line to the anal verge with anal fistula was 19.9 ± 6.0 mm for male and 20.0 ± 5.2 mm for female, respectively. Although the distance from the dentate line to the anal verge in males was slightly shorter than that in females, there was no significant difference between them (*P* = 0.902). Based on the internal opening judgment method, there were 68 male patients with anal fistula and 9 female patients with anal fistula. The distance from the dentate line to the anal verge in male and female patients with anal fistula was 18.3 ± 8.4 mm and 17.1 ± 6.1 mm, respectively. The distance from the dentate line to the anal verge in males was longer than that in females, but significant difference was detected (*P* = 0.026).

### Correlation between body weight and the distance from the dentate line to the anal verge

**Fig. 6:**
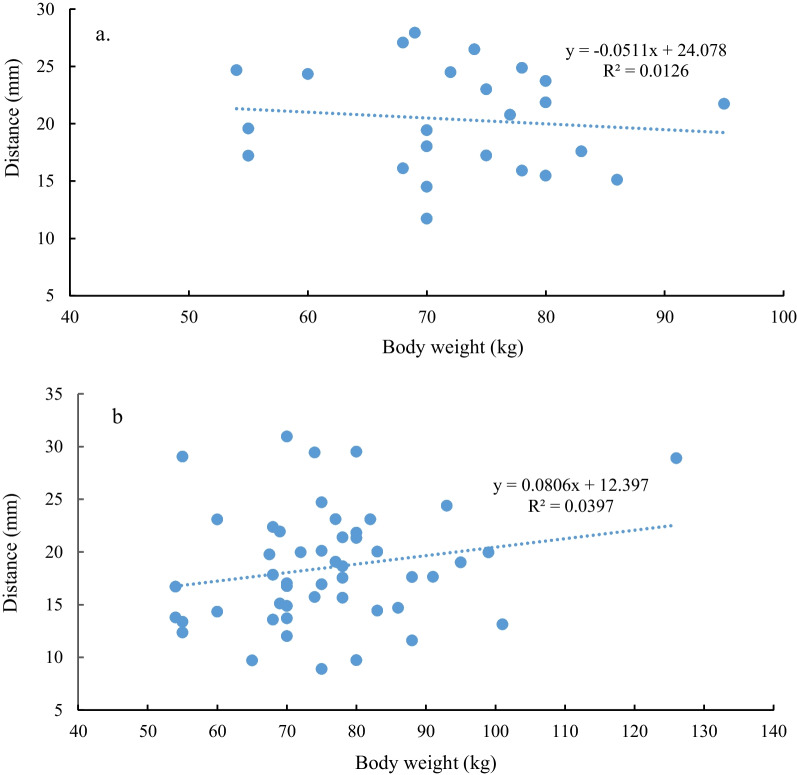
Correlation of body weight with distance from dentate line to anal verge [**a** based on the mucosal judgment method (N = 49); **b** based on the internal opening method (N = 24)]

According to the internal opening judgment method and the mucosa judgment method, there was no statistically significant correlation between the weight of patients with anal fistula and the distance from the dentate line to the anal verge. The Pearson correlation coefficients were 0.199 and 0.112, respectively, and the *P* values were 0.17 and 0.602, respectively (Fig. 6a, b).

### Relationship between the internal opening and the dentate line

We used the distance from the internal opening to the dentate line estimated by surgeons before surgery as the gold standard. Usually, the dynamic contrast-enhanced MRI early arterial phase images can accurately show the position of the internal opening. Based on the morphology of the anal canal mucosa, the accuracy rate of physician 1 in evaluating the positional relationship between internal opening and dentate line was 84.6%, and that of physician 2 was 91.0%. On the oblique coronal T2WI image, we took the 18.2–20 mm distance from the anal verge (the lowest point of the subcutaneous part of external anal sphincter) as the MRI-defined dentate line position, the accuracy rate of physician 1 to determine the positional relationship between the internal opening and the dentate line was 83.3%, and the accuracy rate of physician 2 was 85.9%. According to the morphology of the anal canal mucosa and the distance from the dentate line to the anal verge on MRI (defined as 18.2–20 mm), the accuracy of physician 1 in judging the positional relationship between the internal opening and the dentate line was 85.9%, and the accuracy of physician 2 was 87.2%. Table 6 shows the sensitivity, specificity, and accuracy of the relationship between the internal opening anal fistula and the dentate line as determined by two physicians based on three different methods. Among the three methods mentioned above, the consistency between the two is good, and the κ values were 0.69, 0.78 and 0.80, respectively.

**Table 6:** Diagnosis results for two physicians

	Sensitivity (%)	Specificity (%)	Accuracy (%)	PPV (%)	NPV (%)
According to anal canal mucosa					
Observer 1	91.7	61.1	84.6	88.7	68.8
Observer 2	98.3	70.0	91.0	90.5	93.3
According to position of the internal opening					
Observer 1	84.7	78.9	83.3	92.6	62.5
Observer 2	91.2	71.4	85.9	89.7	75
According to anal canal mucosa and position of the internal opening					
Observer 1	89.7	75	85.9	89.7	71.4
Observer 2	93.1	70	87.2	90	77.8

*PPV* Positive predictive value, *NPV* Negative predictive value

According to the mucosal judgment method, the internal opening judgment method and the combination of the two methods, the research objects with the same evaluation results by two physicians were compared with the surgical results again. The results are shown in Table 7. Among the three methods, the consistency of MRI evaluation was good, and κ values were 0.70, 0.63 and 0.78, respectively.

**Table 7:** Diagnosis results for MRI

	Sensitivity (%)	Specificity (%)	Accuracy (%)	PPV (%)	NPV (%)
Mucosal method	96.2	68.8	89.9	91.1	84.6
Internal opening method	92.2	68.4	85.7	88.7	76.5
Combination of two method	94.3	83.3	91.5	94.3	83.3

## Discussion

Dentate line is useful for the evaluation of diseases of the anal canal such as lower rectal cancer, anal canal cancer, anal fistula, hemorrhoids [3, 6, 9, 10]. Generally, above the dentate line, lymph can drain to the perirectal lymph nodes, pudendal lymph nodes and along the inferior mesenteric artery to the internal iliac system, while below the dentate line, lymph can drain to the inguinal, femoral and external iliac lymph nodes [11]. The clinician can determine whether the tumor has invaded the dentate line based on lymph node metastasis. Anorectal doctors need to consider whether rectal cancer invades the dentate line when formulating a treatment plan for low rectal cancer [12].

Existing research shows that anorectal specialists are crucial in planning the treatment of whether low rectal cancer invades the dentate line [12]. The internal opening of the fistula usually originates from the dentate line [13]. When the position of the internal opening of the anal fistula is not on the same horizontal line as the dentate line, the preoperative MRI examination needs to clarify the positional relationship between the internal opening and the dentate line, which is often cared by clinicians. Difficulty in operation is a potential cause of postoperative recurrence in patients with high anal fistula. To reduce the postoperative recurrence rate, the surgical plan of anal fistula usually considers information such as the location and number of internal openings, the direction of the fistula, and the number of branches fistulas [14]. Previous studies have shown that the internal opening of the transsphincteric anal fistula was located above the dentate line, and the angle between the running direction of the fistula and the longitudinal axis of the anal canal was 35° (range 14–91°) [15], but it was difficult to completely remove the internal opening and fistula from this angle during surgery, which increases the recurrence rate after surgery. Therefore, preoperative MRI may provide the surgeon with more important information [16]. Clinical classification of hemorrhoids is mainly based on the anatomical mark of the anal canal dentate line. Hemorrhoids can usually be divided into three categories according to the position of the dentate line of the anal canal, namely internal hemorrhoids (above the dentate line), external hemorrhoids (below the dentate line), and mixed hemorrhoids (the location of varicose veins crosses the dentate line of the anal canal) [10]. Different types of hemorrhoids had different histology and clinical symptoms, and surgeons should treat differently. The prognosis and treatment options and effects of hemorrhoids mainly depended on its type [11, 17, 18].

The dentate line is the most important anatomical landmark of the anal canal, but it is difficult to directly identify it on MRI [19]. In this study, according to the location of the internal opening of the anal fistula, the MRI-defined dentate line was located 18.2 ± 8.1 mm above the anal verge, of which 18.3 ± 8.4 mm (S.D.) for men and 17.1 ± 6.1 mm (S.D.) for women, respectively. According to the morphology of the anal canal mucosa, the MRI-defined dentate line was located 20.0 ± 5.3 mm (S.D.) above the anal verge, of which 19.9 ± 6.0 mm (S.D.) for men and 20.0 ± 5.2 mm (S.D.) for women, respectively. Our results showed that the distance between the dentate line in female and the anal verge was slightly longer than that of males, which is slightly lower than the distance determined by histoanatomy of the anal canal (2.2 cm for men and 2.0 cm for women) [20]. According to the morphological characteristics of the anal canal mucosa and the MRI-defined dentate line, we evaluated the relationship between the internal opening and the dentate line in 70 patients with anal fistula on MRI. The accuracy of the three methods was over 80%, and the results were similar to a previous study [21].

The anal canal is composed of mucosa, submucosa, internal sphincter, longitudinal muscle and external sphincter [22]. It is generally believed that, above the dentate line, the mucosal layer is thin and folded, the T2-weighted image shows a high signal, and the mucus secreted by the mucosal layer also shows a high signal. There was no mucosal layer below the dentate line, and it was covered with squamous epithelium [4]. Therefore, in the smooth anal cavity, no wrinkled, hypertrophic mucosa can be seen on the T2-weighted image. The lack of mucus-secreting epithelium below the level of the dentate line, shown by periodic acid-Schiff stain, so the mucosal structure cannot be seen on T2WI [23]. The dentate line was the transition zone from the glandular mucosa to the squamous epithelium [4]. Based on this finding, in the early dynamic contrast-enhanced MRI image of the oblique transverse position, the irregular and fattened mucosa in the anal canal was determined as the position of the MRI-defined dentate line [21]. Radiological diagnosis experience shows that dynamic contrast-enhanced MRI can better distinguish the anal canal epithelium morphologically [21, 24]. There are wrinkled mucosa and mucus above the dentate line, in the early arterial phase of dynamic contrast-enhanced MRI, the mucosa is obviously enhanced in the form of thin lines, which can be clearly displayed on the image [21]. The anal luminal surface below the dentate line is covered with squamous epithelium, in the arterial phase, it was manifested as thin line-like obvious enhancement. We think that this may be caused by the mucus secreted by the mucous membrane on the dentate line flowing to the level below the dentate line, or by the thin mucous membrane covering the anal cavity with no secretory function. Based on this phenomenon, this study selected patients with anal fistula, recorded the distance between the internal opening and the dentate line during the operation, and calculated the distance between the dentate line and the anal verge on MRI using a simple formula. Results demonstrate that if the distance between the internal opening and the dentate line in fistula patients was recorded, a simple formula would be used to indirectly calculate the distance between the dentate line and the anal verge on MRI.

Our research has three limitations. First, the dentate line determined by the surgeon during the operation does not correspond exactly to the result identified on the MRI image. During the operation, the surgeon's measurement of the distance between the internal opening and the dentate line is highly subjective, and there may be a certain error with the actual situation. This may limit the objectivity of the dentate line of MRI-defined to a certain extent. Second, the two physicians' judgments on the position of the internal opening and the changes in the morphology of the mucosa on MRI are likely to have diagnostic bias, especially for physicians who have no clinical experience and lack of theoretical knowledge. Third, we set the position of the anal verge at the lowest point of the subcutaneous part of external anal sphincter. Sometimes the lowest point of the subcutaneous part of external anal sphincter on both sides is not in the same plane, one side is high and the other side is low. In this study, the relatively normal side of the subcutaneous part of external anal sphincter may lead to a certain bias in the measurement results.

## Data Availability

The datasets used and/or analyzed during the current study are available from the corresponding author on reasonable request.
